# Pretreatment Neutrophil-to-Lymphocyte Ratio as a Prognostic Biomarker in Unresectable or Metastatic Esophageal Cancer Patients With Anti-PD-1 Therapy

**DOI:** 10.3389/fonc.2022.834564

**Published:** 2022-04-13

**Authors:** Yiming Gao, Zhibo Zhang, Yao Li, Siyuan Chen, Jiangyue Lu, Liangliang Wu, Zhiqiang Ma, Yi Hu, Guoqing Zhang

**Affiliations:** ^1^ Medical School of Chinese People’s Liberation Army (PLA), Beijing, China; ^2^ Department of Oncology, The First Medical Center, Chinese People’s Liberation Army (PLA) General Hospital, Beijing, China; ^3^ Department of Cardiothoracic Surgery, The 78th Group Army Hospital of Chinese People’s Liberation Army (PLA), Mudanjiang, China; ^4^ Harbin Medical University Cancer Hospital, Harbin, China; ^5^ Institute of Oncology, The Fifth Medical Center of Chinese People’s Liberation Army (PLA) General Hospital, Beijing, China

**Keywords:** esophageal cancer, immune checkpoint inhibitor, programmed cell death 1, neutrophil-to-lymphocyte ratio, prognosis

## Abstract

**Background:**

The neutrophil-to-lymphocyte ratio (NLR) is an inflammatory index calculated by the absolute neutrophil count dividing the absolute lymphocyte count, and its prognostic role in esophageal cancer (EC) patients with anti-PD-1 therapy remains unclear.

**Methods:**

A total of 140 unresectable or metastatic EC patients receiving PD-1 inhibitor treatment were included from Jan 2016 to Mar 2020. Kaplan–Meier method and log-rank test were used for comparing overall survival (OS) and progression-free survival (PFS) between groups. Multivariate Cox analysis was performed to assess the prognostic value of NLR.

**Results:**

The cutoff value of NLR was set at 5, and the median follow-up time was 20.0 months. Patients with pretreatment NLR <5 had higher ORR (46.7% vs. 12.1%; *p* < 0.001) and DCR (85.0% vs. 69.7%; *p* = 0.047) than those with NLR ≥5. Kaplan–Meier curves showed that pretreatment NLR <5 was associated with longer PFS (median: 10.0 vs. 3.5 months, *p* < 0.0001) and OS (median: 22.3 vs. 4.9 months, *p* < 0.0001). Multivariate analysis demonstrated that pretreatment NLR ≥5 independently and significantly increased the risk of disease progression (hazard ratio (HR), 1.77 (95% confidence interval (CI), 1.12–2.82); *p* = 0.015) and death (HR, 4.01 (95% CI, 2.28–7.06); *p* < 0.001). Subgroup analysis showed that pretreatment NLR ≥5 was associated with poor efficacy and survival in most subsets.

**Conclusions:**

Our findings showed that pretreatment NLR was independently and significantly associated with the efficacy and prognosis of EC patients treated with PD-1 inhibitors. NLR could serve as a convenient and useful prognostic biomarker for EC patients with anti-PD-1 therapy.

## Introduction

Esophageal cancer (EC) continues to be the top 10 most common tumor types and one of the leading causes of cancer-related deaths worldwide, which seriously threatens human health (1). Currently, systemic chemotherapy and targeted therapy are the primary treatments of unresectable or metastatic EC in clinical practice. First-line systemic chemotherapy, such as fluoropyrimidine combined with oxaliplatin or cisplatin, has been recommended for locally advanced or metastatic EC, however, the prognosis remains poor with the median survival time of around 1 year (2). As a new type of approach, targeted therapy such as trastuzumab [a monoclonal antibody against human epidermal growth factor receptor-2 (HER-2)] plus chemotherapy has been recommended for patients with HER2-positive metastatic esophageal adenocarcinoma. However, limited patients are available for targeted therapy, and patient response rates are 30%–60% (2–6). Patients with early-stage EC can be treated with surgical resection, but most patients were unresectable or metastases at diagnosis (7). Despite development in the treatment, the 5-year survival is still poor at below 20% (8, 9).

Immune checkpoints, represented by programmed cell death-1 (PD-1) and cytotoxic T-lymphocyte-associated protein-4 (CTLA-4), are inhibitory regulators in the immune system, facilitating the maintenance of peripheral tolerance and preventing autoimmunity (10). PD-1 expresses increasingly on the surface of activated T cells, while tumor cells could induce immune suppression by upregulating its ligand PD-L1 expression, and the combination of PD-1 and PD-L1 could inhibit the antitumor effect of T cells (11). Unlike the antitumor mechanism of traditional chemotherapy, immune checkpoint inhibitors (ICIs) could suppress tumor progression by enhancing the efficacy and specificity of T cells (12, 13). Blocking the PD-1/PD-L1 pathway has shown great benefit in various cancers; however, not all patients could get sustained benefits from immunotherapy (14). Several molecular and genomic biomarkers have been studied to show predictive value for immunotherapy in multiple cancer types, including PD-L1 expression, tumor mutational burden (TMB), and microsatellite instability status (15, 16). However, these biomarkers have not been widely used in clinical practice due to their limitations, such as requirements for eligible organizations, willingness for repeated biopsy of patients, tedious follow-up sequencing analysis, and unrecognized unified standard quantification (17). Therefore, it is of crucial importance to identify biomarkers to guide the use of ICIs.

Tumor-associated inflammation plays a critical role in the development of cancer, promoting tumor progression and influencing the host immune responses (18–21). The neutrophil-to-lymphocyte ratio (NLR), defined as absolute neutrophil counts divided by lymphocyte counts, may represent a balance between a protumor inflammatory state and an antitumor immune response. Previous studies have shown the prognostic value of NLR in various cancers, such as nonsmall cell lung cancer, breast cancer, and melanoma (22–25). However, no studies regarding EC patients treated with PD-1 inhibitors have been reported. Therefore, we conducted this study to investigate whether pretreatment NLR was associated with the efficacy and prognosis of unresectable or metastatic EC patients with anti-PD-1 therapy.

## Methods

### Study Design and Patients

This real-world study was conducted in the Chinese PLA General Hospital (Beijing, China). Patients with EC receiving immune checkpoint inhibitors (ICIs) were detected from Jan 2016 to Mar 2021. Included patients should meet the following criteria: (1) EC was diagnosed by pathology; (2) patients with unresectable or metastatic EC; (3) patients were agreed to the treatment plan and received ICI treatment. Patients were excluded following the criteria: (1) patients received ICI treatment less than 2 cycles; (2) patients had no imaging data for evaluating treatment efficacy; (3) patients have no blood test results at baseline (within 1 week before initial ICI treatment). The study was performed following the ethical standards of the Chinese PLA General Hospital and conducted by the Declaration of Helsinki.

### Data Extraction

Two investigators (YG and ZZ) independently performed data extraction, including age, sex, stage, distant metastasis, histological type, smoking history, ICI drugs, Eastern Cooperative Oncology Group Performance Status (ECOG PS), prior operation, treatment lines, treatment type, treatment efficacy, and pretreatment blood test results of neutrophil count and lymphocyte count. Any disagreement was resolved by the third investigator (GZ). The cutoff value of NLR was set at 5, which was calculated by X-tile software based on data (26). Treatment efficacy was defined as complete response (CR), partial response (PR), stable disease (SD), or progressive disease (PD) according to the Response Evaluation Criteria in Solid Tumors version 1.1 (RECIST V1.1). The objective response rate (ORR) was the percentage of patients with CR and PR. Disease control rate (DCR) was the percentage of patients with CR, PR, and SD. Progression-free survival (PFS) was defined as the interval time from ICI start to disease progression or death (which occurred first). Overall survival (OS) was the interval time from ICI start to death. All patients were followed up by searching medical records and counseling telephone every 3 months. The cutoff date was Sep. 30, 2021.

### Statistical Analysis

Statistical analyses were performed using IBM SPSS (version 19.0), and graphs were drawn with R version 4.1.0 using packages of survival (version 3.2-11), ggplot2 (version 3.3.5), and forestplot (version 1.10.1). X-tile 3.6.1 was used to determine the cutoff value of NLR. Kaplan–Meier method was used for analyzing PFS and OS, and survival curves were compared by log-rank test. Chi-square or Fisher’s exact test was used for comparing categorical variables. Hazard ratio (HR) and 95% confidence interval (CI) were calculated by Cox proportional-hazard regression model. Univariate and multivariate analyses were performed to determine the independent prognostic factors. All statistical tests were bilateral, and *p* < 0.05 was considered to be statistically significant.

## Results

### Study Population

**Figure 1:**
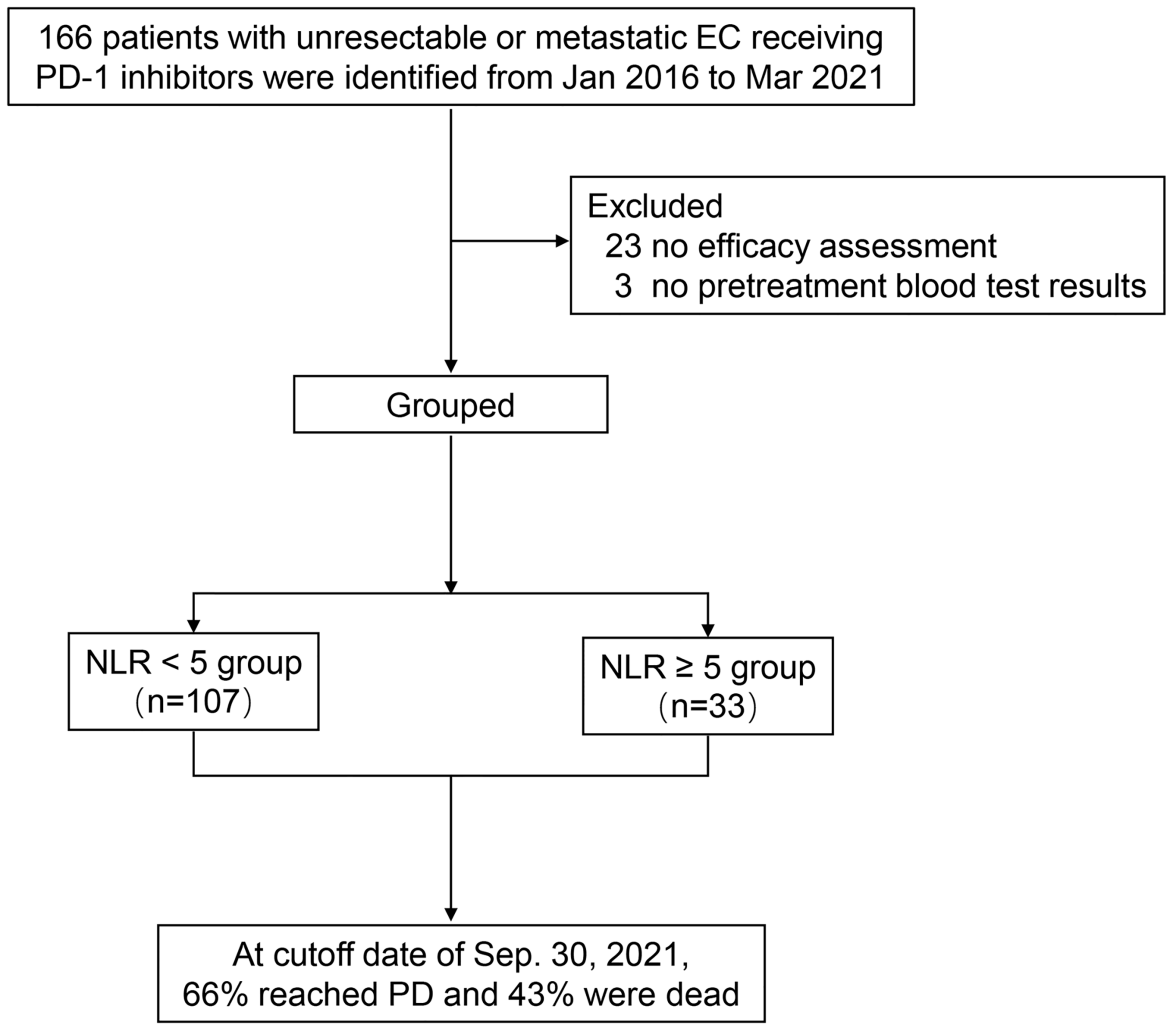
Diagram of the study.

A total of 166 consecutive patients with unresectable or metastatic esophageal cancer receiving ICIs were identified at first, of which 23 patients had no efficacy assessment, and 3 patients had no pretreatment blood test results. Finally, 140 patients were included for data analysis (**Figure 1**). Patients’ clinical data are summarized in **Table 1**. The median age was 60 years (range: 40–80). Of 140 patients in this cohort, 91.4% were men, 70% had distant metastasis, 92.9% with squamous carcinoma, 67.1% had smoking history, 92.9% with ECOG PS of 0–1, and 21.4% were postoperative recurrence; most patients (82.9%) received ICI combination therapy, including 75% with chemotherapy and 7.9% with target vascular endothelial grow factor (VEGF) therapy. PD-1 inhibitor of pembrolizumab, toripalimab, nivolumab, sintilimab, and camrelizumab accounted for 42.9%, 21.4%, 17.9%, 12.1%, and 5.7%, respectively. Of patients, 55% received ICIs at 1-line treatment, 30.7% at 2-line treatment, and 14.3% at ≥3-line treatment; 54 patients (38.6%) were evaluated PR, 60 (42.9%) were SD, and the remaining 26 (18.6%) were PD. The median value of NLR was 3.18 in the range of 0.94 to 89.7, and most patients (76.4%) were with NLR <5. The median follow-up time was 20 months with 95% CI of 15 to 25 months.

**Table 1:** Characteristics of included patients.

Characteristics	No. of patients (*N* = 140)	Percentage (%)
Age [year; median (range)]	60 (40−80)	–
<70	117	83.6
≥70	23	16.4
Sex		
Men	128	91.4
Women	12	8.6
Stage		
I	2	1.4
II	5	3.6
III	7	5.0
IV	71	50.7
Unknown	55	39.3
Distant metastasis		
No	42	30.0
Yes	98	70.0
Histological type		
Squamous	130	92.9
Adenocarcinoma	4	2.9
Unknown	6	4.3
Smoking history		
Never	46	32.9
Current/former	94	67.1
PD-1 inhibitors		
Pembrolizumab	60	42.9
Toripalizumab	30	21.4
Nivolumab	25	17.9
Sintilimab	17	12.1
Camrelizumab	8	5.7
ECOG PS		
0–1	130	92.9
≥2	10	7.1
Prior operation		
No	110	78.6
Yes	30	21.4
Treatment lines		
1 line	77	55.0
2 lines	43	30.7
≥3 lines	20	14.3
Treatment type		
ICI monotherapy	24	17.1
ICI combination therapy	116	82.9
+ Chemotherapy	105	75.0
+ Target VEGF therapy	11	7.9
Best efficacy		
PR	54	38.6
SD	60	42.9
PD	26	18.6
Pretreatment NLR		
Median (range)	3.18 (0.94−89.7)	
Low (<5)	107	76.4
High (≥5)	33	23.6

ECOG PS, Eastern Cooperative Oncology Group Performance Status; PD-1, programmed cell death 1; CR, complete response; PR, partial response; SD, stable disease; PD, progression disease; NLR, neutrophil-to-lymphocyte ratio.

### Comparing HR Between Groups Using Different NLR Cutoff Values

**Figure 2:**
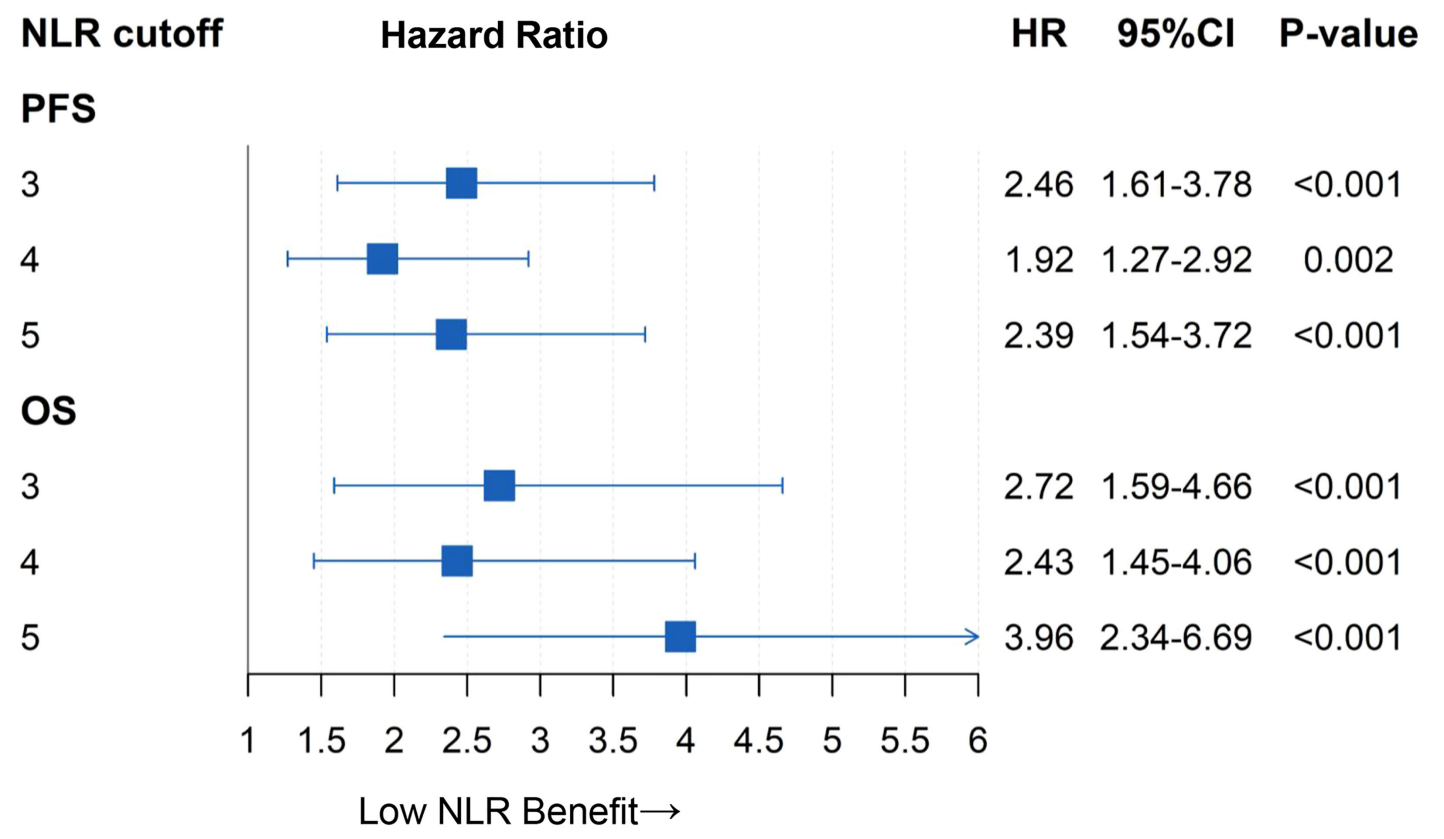
Comparing hazard ratio between two groups using different NLR cutoff values.

Different cutoff values of pretreatment NLR were analyzed in the study. As shown in **Figure 2**, patients with high NLR had more risks of shortened PFS and OS than those with low NLR when the cutoff value of NLR was set at 3 (PFS: HR, 2.46; OS: HR, 2.72; *p* < 0.001), 4 (PFS: HR, 1.92; OS, HR 2.43; *p* = 0.002), and 5 (PFS: HR, 2.39; OS: HR, 3.96; *p* < 0.001), respectively. The cutoff value of 5 was optimal, for the hazard ratio of OS was the highest between the two groups.

### Univariate and Multivariate Analyses of Pretreatment NLR

**Figure 3:**
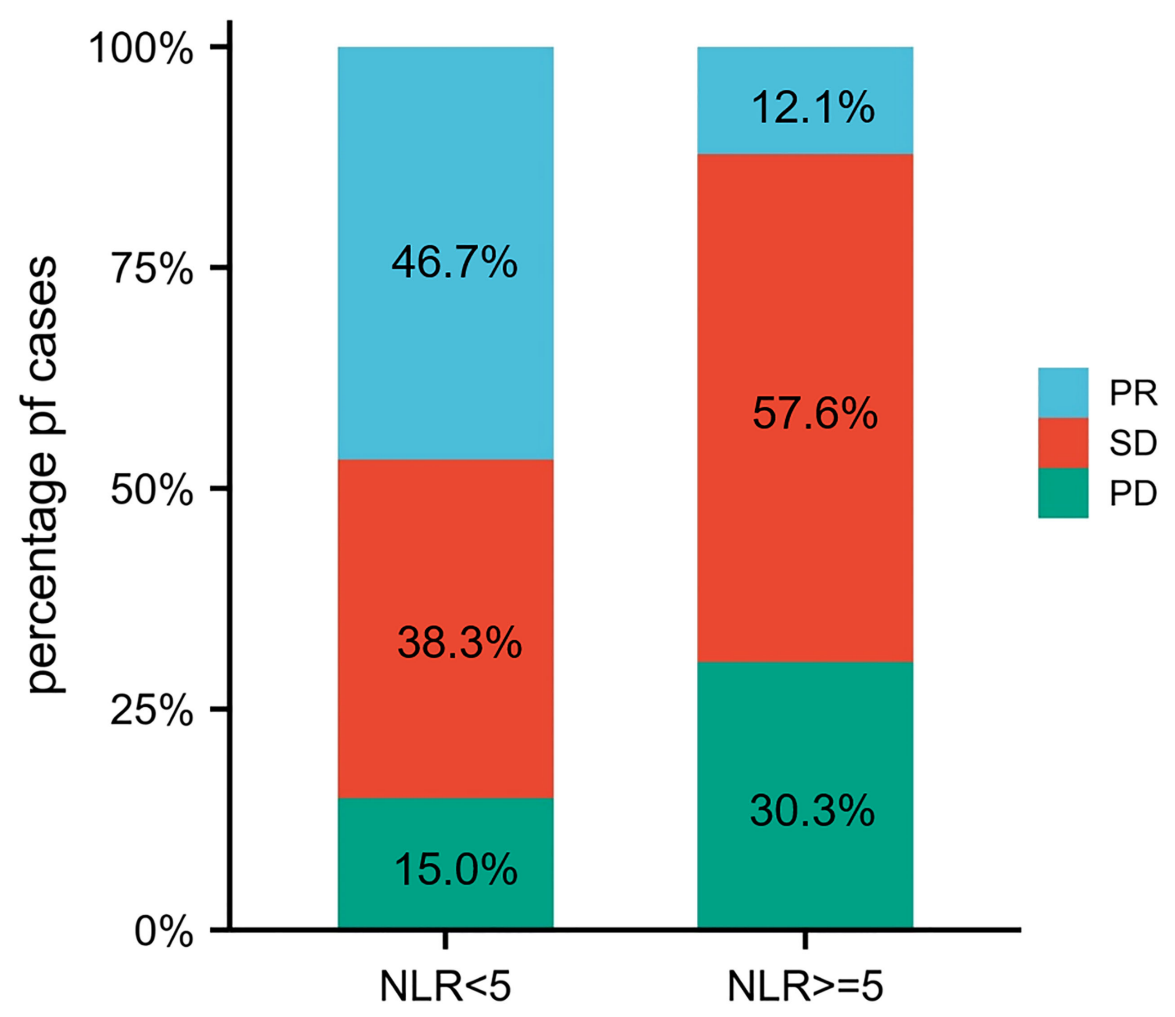
The distribution of treatment efficacy between two groups.

As shown in **Table 2** and **Figure 3**, patients with pretreatment NLR <5 had better ORR (46.7% vs. 12.1%; *p* < 0.001) and DCR (85.0% vs. 69.7%; *p* = 0.047) than those with NLR ≥5. As demonstrated in **Table 3**, pretreatment NLR <5 was associated with longer PFS (median: 10.0 vs. 3.5 months; HR, 0.42 (95% CI, 0.27–0.65); *p* < 0.0001) compared with pretreatment NLR ≥5. Univariate Cox regression analysis showed that treatment lines, distant metastasis, ECOG PS, treatment type, and pretreatment NLR were associated with PFS in patients with EC receiving anti-PD-1 therapy (*p* < 0.05). After multivariate Cox regression analysis, the results showed that pretreatment NLR ≥5 independently and significantly increased the risk of disease progression (HR, 1.77 (95% CI, 1.12–2.82); *p* = 0.015), Post lines of therapy (≥3 lines: HR, 2.74 [95% CI, 1.44–5.22], p = 0.002; ≥2 lines: HR, 1.77 [95% CI, 1.02–3.07]; p = 0.043), ECOG PS ≥2 (HR, 2.95 [95% CI, 1.43–6.11]; p = 0.004), and anti-PD-1 monotherapy (HR, 1.89 [95% CI, 1.07–3.23]; p = 0.025) were independently associated with worse OS.

**Table 2:** Comparing treatment efficacy between two groups.

	NLR <5	NLR ≥5	*p*-value
Treatment efficacy [*n* (%)]			
CR	0 (0)	0 (0)	–
PR	50 (46.7)	4 (12.1)	–
SD	41 (38.3)	19 (57.6)	–
PD	16 (15.0)	10 (30.3)	–
ORR	50 (46.7)	4 (12.1)	<0.001
DCR	91 (85.0)	23 (69.7)	0.047

CR, complete response; PR, partial response; SD, stable disease; PD, progression disease; ORR, objective response rate; DCR, disease control rate; NLR, neutrophil-to-lymphocyte ratio.

**Table 3:** Univariate and multivariate analyses for PFS and OS.

Variable	Category	PFS				OS			
		Univariate analysis		Multivariate analysis		Univariate analysis		Univariate analysis	
		HR (95% CI)	*p*-value	HR (95% CI)	*p*-value	HR (95% CI)	*p*-value	HR (95% CI)	*p*-value
Age (year)	<70	Reference	–	–	–	Reference	–	–	–
	≥70	0.55 (0.28–1.05)	0.07	–	–	0.60 (0.26–1.39)	0.233	–	–
Sex	Men	Reference	–	–	–	Reference	–	–	–
	Women	0.86 (0.42–1.78)	0.685	–	–	0.59 (0.21–1.64)	0.314	–	–
Smoking history	No	Reference	–	–	–	Reference	–	–	–
	Yes	0.79 (0.51–1.20)	0.268	–	–	0.89 (0.52–1.52)	0.66	–	–
Histology	Squamous	Reference	0.98	–	–	Reference	0.88	–	–
	Adenocarcinoma	0.99 (0.36–2.71)	0.84	–	–	0.79 (0.25–2.56)	0.73	–	–
	Unknown	1.12 (0.25–5.02)	0.99	–	–	1.02 (0.17–6.13)	0.70	–	–
Treatment lines	1 line	Reference	<0.001	Reference	0.008	Reference	<0.001	Reference	0.011
	2 lines	2.85 (1.80–4.51)	<0.001	1.77 (1.02–3.07)	0.043	2.43 (1.35–4.37)	0.003	1.16 (0.56–2.41)	0.694
	≥3 lines	4.21 (2.38–7.46)	<0.001	2.74 (1.44–5.22)	0.002	4.46 (2.27–8.75)	<0.001	2.92 (1.36–6.30)	0.006
Stage	I	Reference	–	–	–	Reference	0.475	–	–
	II	0.97 (0.23–4.05)	0.961	–	–	0.54 (0.07–4.00)	0.543	–	–
	III	0.44 (0.11–1.82)	0.256	–	–	0.42 (0.06–3.14)	0.401	–	–
	IV	1.46 (0.57–3.76)	0.434	–	–	2.07 (0.71–6.06)	0.184	–	–
	Unknown	1.09 (0.71–1.69)	0.688	–	–	0.88 (0.52–1.50)	0.635	–	–
Distant metastasis	No	Reference	–	Reference	–	Reference	–	Reference	–
	Yes	2.31 (1.36–3.93)	0.002	1.25 (0.69–2.26)	0.47	2.12 (1.07–4.19)	0.032	0.93 (0.43–1.99)	0.842
ECOG PS	0–1	Reference	–	Reference	–	Reference	–	Reference	–
	≥2	5.23 (2.63–10.41)	<0.001	2.95 (1.43–6.11)	0.004	12.11 (5.63–16.09)	<0.001	10.59 (4.30–26.06)	<0.001
Treatment type	Combination therapy	Reference	–	Reference	–	Reference	–	Reference	–
	Monotherapy	3.33 (0.10–0.38)	<0.001	1.89 (1.07–3.23)	0.025	3.13 (1.79–5.56)	<0.001	2.00 (1.02–4.00)	0.048
Prior operation	No	Reference	–	–	–	Reference	–	–	–
	Yes	1.31 (0.82–2.09)	0.265	–	–	1.34 (0.76–2.34)	0.313	–	–
Pretreatment NLR	Low (<5)	Reference	–	Reference	–	Reference	–	Reference	–
	High (≥5)	2.39 (1.53–3.72)	<0.001	1.77 (1.12–2.82)	0.015	3.96 (2.34–6.69)	<0.001	4.01 (2.28–7.06)	<0.001

PFS, progression-free survival; OS, overall survival; ECOG PS, Eastern Cooperative Oncology Group Performance Status; HR, hazard ratio; CI, confidence interval.

**Figure 4:**
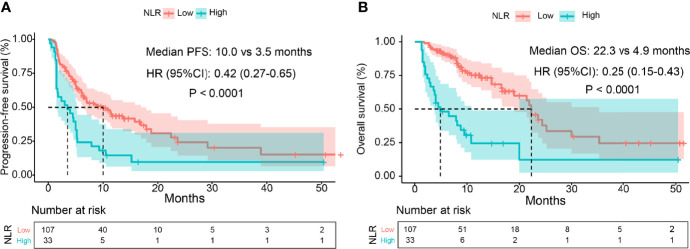
Kaplan-Meier curves of PFS and OS. Part **(A)** is the curve of PFS and part **(B)** is the curve of OS.

As shown in **Figure 4**, pretreatment NLR <5 was correlated with longer OS (median: 22.3 vs. 4.9 months; HR, 0.25 (95% CI, 0.15–0.43); *p* < 0.0001) compared with NLR ≥5. Univariate Cox regression analysis showed that treatment lines, distant metastasis, ECOG PS, treatment type, and pretreatment NLR were associated with OS in patients with EC receiving ICIs (*p* < 0.05). After multivariate Cox regression analysis, the results showed that pretreatment NLR ≥5 independently and significantly increased the risk of death (HR, 4.01 (95% CI, 2.28–7.06); *p* < 0.001), so as for ≥3-line treatment (HR, 2.92 (95% CI, 1.36–6.30); *p* = 0.006), ECOG PS ≥2 (HR, 10.59 (95% CI, 4.30–26.06); *p* < 0.001), and anti-PD-1 monotherapy (HR, 2.00 (95% CI, 1.02–4.00); *p* = 0.048), respectively (**Table 3**).

### Subgroup Analysis of Pretreatment NLR

**Figure 5:**
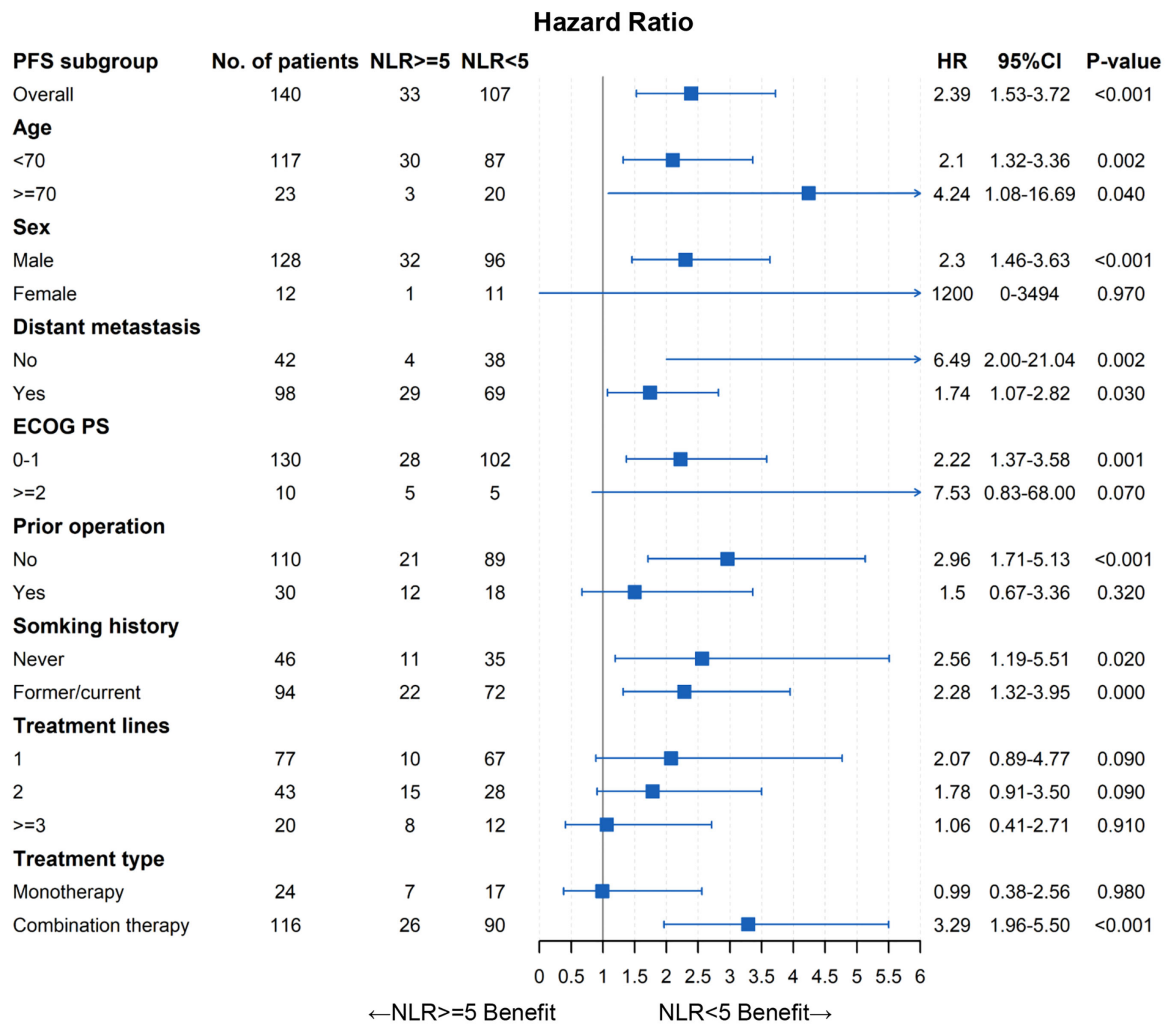
Forest plot of PFS.

**Figure 6:**
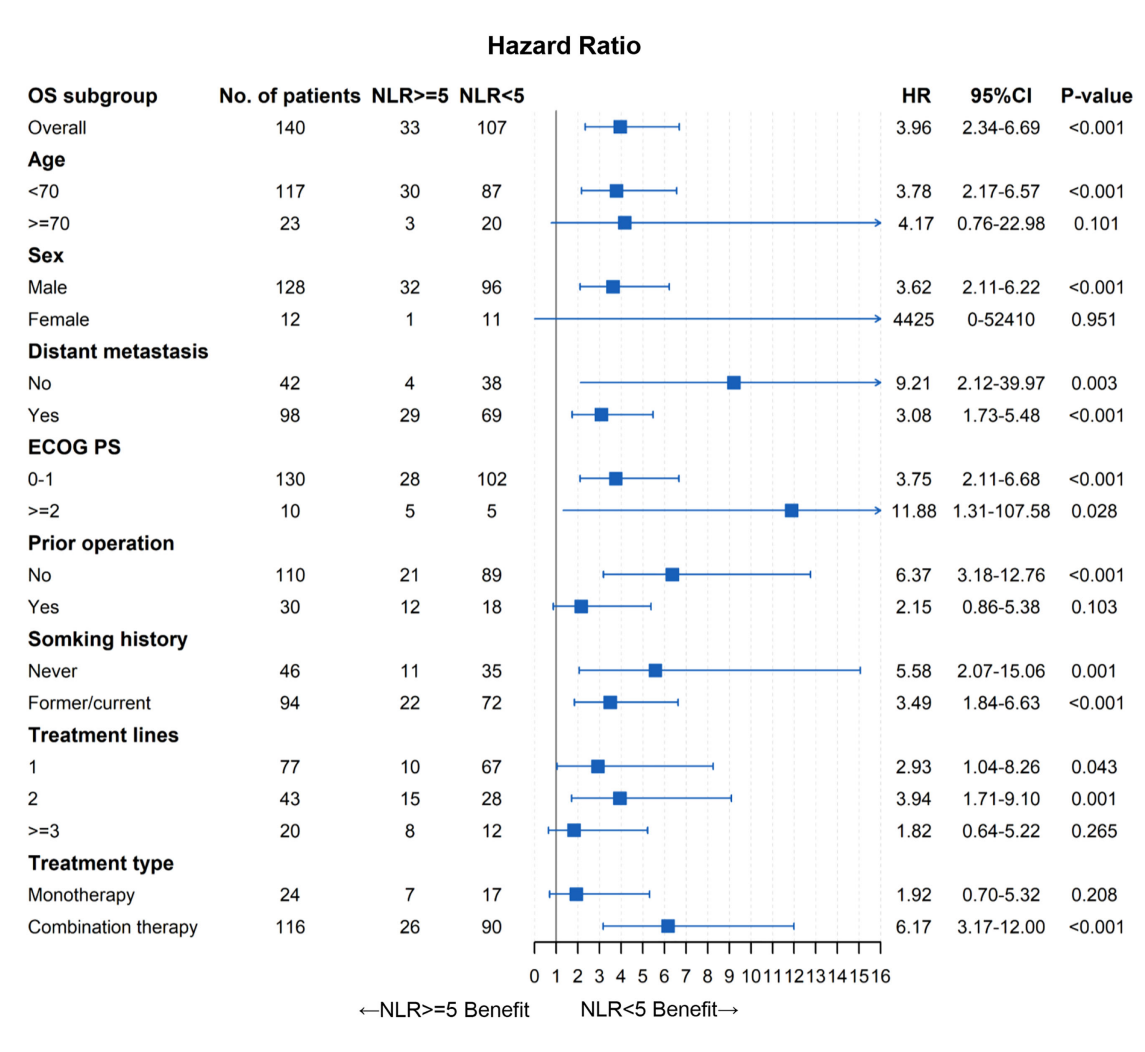
Forest plot of OS.

As shown in **Table 4**, there were more patients with distant metastasis, prior operation, or ≥3-line treatment in pretreatment NLR ≥5 than those with NLR <5 (*p* < 0.05). Thus, subgroup analysis stratified by patients’ characteristics was conducted to further evaluate the prognostic value of pretreatment NLR. As demonstrated in **Figures 5**, **6**, pretreatment NLR <5 was significantly associated with better PFS and OS in most subsets. However, there was no significance in subgroups of women, ECOG PS ≥2, prior operation, treatment lines (1, 2, ≥3), and anti-PD-1 monotherapy for PFS (*p* > 0.05) and subgroups of age ≥70, women, prior operation, treatment lines ≥3, and anti-PD-1 monotherapy for OS (*p* > 0.05).

**Table 4:** Differences of patients’ characteristics between two groups.

Characteristics	NLR <5	NLR ≥5	*p*-value
Age (year)			
<70	87	30	0.283
≥70	20	3	
Sex			
Men	96	32	0.294
Women	11	1	
Distant metastasis			
No	38	4	0.010
Yes	69	29	
Histological type			
Squamous	98	32	0.479
Adenocarcinoma	4	0	
Unknown	5	1	
Smoking history			
Never	35	11	1.000
Current/former	72	22	
ECOG PS			
0–1	102	28	0.056
≥2	5	5	
Prior operation			
No	89	21	0.027
Yes	18	12	
Treatment lines			
1	67	10	0.004
2	28	15	
≥3	12	8	
Treatment type			
ICI monotherapy	17	7	0.597
ICI combination therapy	90	26	

ECOG PS, Eastern Cooperative Oncology Group Performance Status; NLR, neutrophil-to-lymphocyte ratio.

## Discussion

EC is one of the most lethal cancers worldwide. The most common subtypes of EC were esophageal squamous cell carcinoma (ESCC) and esophageal adenocarcinoma (EAC), and ESCC accounts for most of EC (90%) (27). In the past decades, the development of treatments in EC had minimal improvements in survival (28). EC remains a frustrating disease with limited therapeutic choices and a poor prognosis. Currently, immunotherapy using ICIs has been considered an important therapeutic strategy with durable antitumor activity in various types of cancers including EC (29–33). Currently, pembrolizumab and nivolumab (PD-1 inhibitor) have been approved for EC treatment in clinical settings, and several trials targeting ICI therapy in advanced EC are ongoing (34, 35). However, it should be noted that most patients could not experience survival benefits. Biomarkers, such as PD-L1 and TMB, have limited predictive value for the unavailability of tumor tissue (36, 37). Therefore, it remains important to explore biomarkers of identifying EC patients who could respond to anti-PD-1 therapy.

Inflammation contributes to the development and progression of cancer; increasing evidence showed that inflammation was associated with the progression of cancer and survival of patients (38). NLR is considered an indicator of systemic inflammation (39). Unlike traditional chemotherapy and radiotherapy, anti-PD-1 therapy is widely used in recent years. Despite the former study investigating the prognostic value of NLR in patients with locally advanced EC receiving definitive chemoradiation therapy (40), no studies are reported on investigating the association between pretreatment NLR and EC treated with PD-1 inhibitors.

To our knowledge, this was the first study to comprehensively evaluate the prognostic value of pretreatment NLR in unresectable or metastatic EC patients with anti-PD-1 therapy. Our findings showed that pretreatment NLR was significantly associated with PFS and OS in EC patients with anti-PD-1 therapy. Multivariate analysis demonstrated that pretreatment NLR was an independent prognostic factor for PFS and OS. We further conducted subgroup analysis stratified by patients’ characteristics, and the results also showed that pretreatment high NLR was associated with worse clinical outcomes in most subgroups. However, there was no significance in some subgroups such as women, prior operation, and anti-PD-1 monotherapy. The following reasons should be taken into consideration: (1) sample sizes of these insignificant subgroups were relatively small, which may result in statistical insignificance. (2) NLR is an inflammatory index closely related to patients’ characteristics such as tumor distant metastasis, prior operation, and poster lines of therapy; therefore, the prognostic value of NLR may be weakened in these subgroups. Despite the above heterogeneity, our findings still revealed that NLR could serve as a convenient and useful prognostic biomarker in EC patients with anti-PD-1 therapy. These results need to be confirmed by further research and investigation.

There were several limitations in the study. Firstly, for the retrospective nature of the study with limited patients, selective bias was inevitable and some confounding factors (such as PD-L1 and TMB) were not analyzed due to the unavailability of data. To avoid the impact of bias as much as possible, multivariate and subgroup analyses were performed, and these results were consistent. Secondly, patients received different PD-1 inhibitors and chemotherapy regimens in the study, which may affect the final results. Thirdly, the cutoff value of NLR was set at 5, which may not be optimal. Lastly, the follow-up was short term, and some patients did not reach endpoints (33.6% did not reach PD and 57.1% did not reach death); therefore, long-term follow-up is still needed. Nevertheless, our study offered a simple and effective biomarker for guiding the application of PD-1 inhibitors in unresectable or metastatic EC patients.

## Conclusion

Our findings showed that pretreatment NLR was independently and significantly associated with the efficacy and prognosis of unresectable or metastatic EC patients treated with PD-1 inhibitors. NLR could serve as a convenient and useful biomarker for identifying patients who can benefit from PD-1 inhibitors. Further prospective studies are warranted to validate these results.

## Data Availability Statement

The raw data supporting the conclusions of this article will be made available by the authors, without undue reservation.

## Ethics Statement

The studies involving human participants were reviewed and approved by the ethics committee of the Chinese PLA General Hospital. The patients/participants provided their written informed consent to participate in this study.

## Author Contributions

GZ conceived the idea of this article. YG completed the work of acquisition of data. ZZ shared the task of data analyzing and manuscript writing. All authors participated in revising the manuscript. All authors listed have made a substantial, direct, and intellectual contribution to the work and approved it for publication.

## Conflict of Interest

The authors declare that the research was conducted in the absence of any commercial or financial relationships that could be construed as a potential conflict of interest.

## Publisher’s Note

All claims expressed in this article are solely those of the authors and do not necessarily represent those of their affiliated organizations, or those of the publisher, the editors and the reviewers. Any product that may be evaluated in this article, or claim that may be made by its manufacturer, is not guaranteed or endorsed by the publisher.

## Abbreviations

EC, esophageal cancer; NLR, neutrophil-to-lymphocyte ratio; PD-1, programmed cell death 1; PD-L1, programmed cell death ligand-1; ECOG PS, Eastern Cooperative Oncology Group Performance Status; CR, complete response; PR, partial response; SD, stable disease; PD, progression disease; ORR, objective response rate; DCR, disease control rate; PFS, progression-free survival; OS, overall survival; HR, hazard ratio; CI, confidence interval; ICIs, immune checkpoint inhibitors; HER-2, human epidermal growth factor receptor-2; VEFR, vascular endothelial grow factor; TMB, tumor mutational burden.
